# A universal neocortical mask for Centiloid quantification

**DOI:** 10.1002/dad2.12457

**Published:** 2023-07-21

**Authors:** Pierrick Bourgeat, Vincent Doré, Christopher C. Rowe, Tammie Benzinger, Duygu Tosun, Manu S. Goyal, Pamela LaMontagne, Liang Jin, Michael W. Weiner, Colin L. Masters, Jurgen Fripp, Victor L. Villemagne, for the Alzheimer's Disease Neuroimaging Initiative, OASIS3, and the AIBL research group

**Affiliations:** ^1^ Australian eHealth Research Centre CSIRO Health and Biosecurity Brisbane Queensland Australia; ^2^ Department of Molecular Imaging & Therapy Austin Health Melbourne Victoria Australia; ^3^ The Florey Institute of Neuroscience and Mental Health University of Melbourne, Parkville Melbourne Victoria Australia; ^4^ Mallinckrodt Institute of Radiology Washington University School of Medicine St. Louis Missouri USA; ^5^ San Francisco Veterans Affairs Medical Center San Francisco California USA; ^6^ Department of Radiology and Biomedical Imaging University of California, San Francisco San Francisco California USA; ^7^ Department of Neurology Washington University School of Medicine St. Louis Missouri USA; ^8^ Department of Psychiatry The University of Pittsburgh Pittsburgh Pennsylvania USA

**Keywords:** amyloid positron emission tomography, Centiloid, Florbetaben, Florbetapir, Flutemetamol, NAV4694, PiB

## Abstract

**INTRODUCTION:**

The Centiloid (CL) project was developed to harmonize the quantification of amyloid beta (Aβ) positron emission tomography (PET) scans to a unified scale. The CL neocortical mask was defined using ^11^C Pittsburgh compound B (PiB), overlooking potential differences in regional distribution among Aβ tracers. We created a universal mask using an independent dataset of five Aβ tracers, and investigated its impact on inter‐tracer agreement, tracer variability, and group separation.

**METHODS:**

Using data from the Alzheimer's Dementia Onset and Progression in International Cohorts (ADOPIC) study (Australian Imaging Biomarkers and Lifestyle + Alzheimer's Disease Neuroimaging Initiative + Open Access Series of Imaging Studies), age‐matched pairs of mild Alzheimer's disease (AD) and healthy controls (HC) were selected: ^18^F‐florbetapir (*N* = 147 pairs), ^18^F‐florbetaben (*N* = 22), ^18^F‐flutemetamol (*N* = 10), ^18^F‐NAV (*N* = 42), ^11^C‐PiB (*N* = 63). The images were spatially and standardized uptake value ratio normalized. For each tracer, the mean AD–HC difference image was thresholded to maximize the overlap with the standard neocortical mask. The universal mask was defined as the intersection of all five masks. It was evaluated on the Global Alzheimer's Association Interactive Network (GAAIN) head‐to‐head datasets in terms of inter‐tracer agreement and variance in the young controls (YC) and on the ADOPIC dataset comparing separation between HC/AD and HC/mild cognitive impairment (MCI).

**RESULTS:**

In the GAAIN dataset, the universal mask led to a small reduction in the variance of the YC, and a small increase in the inter‐tracer agreement. In the ADOPIC dataset, it led to a better separation between HC/AD and HC/MCI at baseline.

**DISCUSSION:**

The universal CL mask led to an increase in inter‐tracer agreement and group separation. Those increases were, however, very small, and do not provide sufficient benefits to support departing from the existing standard CL mask, which is suitable for the quantification of all Aβ tracers.

**HIGHLIGHTS:**

- This study built an amyloid universal mask using a matched cohort for the five most commonly used amyloid positron emission tomography tracers.
- There was a high overlap between each tracer‐specific mask.
- Differences in quantification and group separation between the standard and universal mask were small.
- The existing standard Centiloid mask is suitable for the quantification of all amyloid beta tracers.

## 1 INTRODUCTION

The Centiloid (CL) project is a standardized method to harmonize amyloid beta (Aβ) quantification for positron emission tomography (PET) images. It not only provides a standard processing pipeline, along with standard masks for neocortical retention and reference regions, but also provides a framework to anchor different tracers and processing pipelines to the same reference values. Using the provided neocortical mask, reference region mask, and a set of published transforms, the five most commonly used Aβ PET tracers can be quantified in Centiloids using the statistical parametric mapping (SPM) pipeline.^1^ ^—^ ^5^

Recent advancements in medical imaging technology have enabled the development of more advanced model‐based methods for generating and improving CL quantification such as non‐negative matrix factorization^6^, ^7^, AmyQ^8^, Aβ index,^9^ and amyloid load (Amyloid^IQ^),^10^ which all use a model fitted to the entire image to perform the quantification. These methods use advanced machine learning techniques to improve inter‐tracer agreement, reduce longitudinal variability, and improve group separation. However, these advanced techniques are limited to research settings and most clinical applications and clinical trials still rely on the use of the standard quantification pipelines (SPM or other similar well validated pipelines) and the associated quantification masks given their simplicity and availability.

One of the potential limitations of the standard neocortical mask is that it was defined using a single tracer, ^11^C‐Pittsburgh compound B (PiB), not accounting for potential differences in regional distribution among Aβ PET tracers. While all five most commonly used Aβ tracers have demonstrated high affinity and specificity for fibrillar Αβ in plaques,^11^ and in vitro comparisons have found that all tracers bind to similar binding sites,^12^, ^13^ differences in tracer affinity and degree of non‐specific binding could lead to slight differences in regional distribution. Using ^11^C‐PiB, one of the tracers with the highest affinity and lowest non‐specific binding as a reference to define the cortical areas to be sampled, could potentially result in the inclusion of regions where binding is not detectable using other tracers. This could increase the noise and reduce the specificity of the other Aβ PET tracers.

In this work, we aim to build a new universal neocortical CL mask based on all five Aβ tracers and evaluate its impact on inter‐tracer agreement, tracer variability, and group separation using both cross‐sectional and longitudinal data, compared to the standard CL mask.

## 2 METHODS

### 2.1 Data

Data used in the preparation of this article came from the Alzheimer's Dementia Onset and Progression in International Cohorts (ADOPIC) study, which combines three large longitudinal cohorts, namely the Australian Imaging Biomarkers and Lifestyle (AIBL) study,^14^ the Open Access Series of Imaging Studies (OASIS‐3),^15^ and the Alzheimer's Disease Neuroimaging Initiative (ADNI) database^16^ (adni.loni.usc.edu). The ADNI was launched in 2003 as a public–private partnership, led by Principal Investigator Michael W. Weiner, MD. The primary goal of ADNI has been to test whether serial magnetic resonance imaging (MRI), PET, other biological markers, and clinical and neuropsychological assessment can be combined to measure the progression of mild cognitive impairment (MCI) and early Alzheimer's disease (AD). For up‐to‐date information, see www.adni‐info.org.

Description of the ADOPIC database was previously described in detail.^7^ Briefly, all Aβ PET data and corresponding T1W MRI acquired before December 31, 2020, in AIBL (3315 Aβ PET scans from 1345 participants), ADNI (3516 Aβ PET scans from 1648 participants), and OASIS‐3 (1398 Aβ PET scans from 748 participants) were extracted, for a total of 8229 Aβ PET scans from 3741 participants. AIBL Aβ PET scans were acquired using one of five tracers: ^11^C‐PiB, ^18^F‐florbetapir (FBP), ^18^F‐florbetaben (FBB), ^18^F‐flutemetamol (FLT), ^18^F‐NAV4694 (NAV). ADNI used three tracers (PiB, FBP, FBB) and OASIS3 two (PiB, FBP).

### 2.2 Population selection

Using data from the ADOPIC study, mild AD patients were selected using the following criteria: clinical diagnosis of AD (with AIBL and ADNI using the National Institute of Neurological and Communicative Disorders and Stroke–Alzheimer's Disease and Related Disorders Association criteria for probable AD^17^ and OASIS‐3 using the 2011 National Institute of Aging–Alzheimer's Association criteria for probable AD^18^), Mini‐Mental State Examination (MMSE) between 20 and 24, and above 25 CL to ensure that they have Aβ pathology.^19^ For each identified mild AD patient, an age and sex‐matched healthy control (HC) was identified using the following criteria: scanned with the same tracer, same sex, MMSE > = 28, Clinical Dementia Rating (CDR) = 0 and CL < 15 and age closest to the target AD patient. The scans selected to build the masks were then excluded from the ADOPIC dataset for the subsequent analysis to avoid any bias.

### 2.3 PET analysis

All PET images from the ADOPIC study were smoothed to a uniform 8 mm resolution to reduce the influence of different scanner sharpness on the derived masks. The images were then spatially normalized to the Montreal Neurological Institute template using the standard SPM CL pipeline.^2^ The spatially normalized images were then mirrored to remove any asymmetry. Standardized uptake value ratio (SUVR) normalization was performed using the CL whole cerebellum mask (WCb) as reference region. Mean AD and HC images were then computed for each tracer along with a corresponding difference image (AD–HC). While different thresholds for the difference images could be explored, this was not the primary aim of this work. Instead, each tracer's threshold was defined so that the resulting mask maximizes the overlap with the original CL mask. This was implemented using a Powel optimizer seeking to maximize the Dice similarity score,^20^ which is used as a measure of masks overlap. The Dice similarity score was selected in this application as it is commonly used to optimize segmentation models. Finally, the universal mask was defined as the intersection of all tracer‐specific masks. The universal mask was then used to recalibrate the CL equation for PiB using the Global Alzheimer's Association Interactive Network (GAAIN) PiB dataset of young controls (YC) and mild AD, followed by each tracer using their respective PiB/^18^F‐tracer pairs from the GAAIN dataset.

### 2.4 Evaluation

Paired *t* tests were used to assess differences in MMSE, CDR, age, and CL between the matched HC and AD for each tracer. Cohen *d* was used to compute the corresponding effect size. Chi‐square was used to assess differences in sex distribution. Analysis of variance was used to assess if there were any differences in MMSE, CDR, age, and CL between the HC (and AD) participants selected for each tracer. Similarly, chi‐square was used to assess differences in sex distribution between the HC (and AD) participants selected for each tracer.

**RESEARCH IN CONTEXT**

1. **Systematic Review**: The authors reviewed the literature using traditional (e.g., PubMed) sources and meeting abstracts and presentations. While amyloid beta (Αβ) positron emission tomography (PET) tracers’ affinity and specificity for fibrillar Αβ in plaques have been compared in vitro, there is limited evidence of potential differences in regional distribution in vivo.
2. **Interpretation**: Our results indicate that using a universal neocortical Centiloid mask led to marginal improvements using our chosen metrics, indicating that a universal mask is not required and that the existing standard mask is suitable for the quantification of all Αβ PET tracers.
3. **Future Directions**: While this article only focused on the target region, a similar exploration should be conducted to choose the optimal reference region for each tracer.

The standard and universal masks were first evaluated on the GAAIN dataset in terms of inter‐tracer correlation using the coefficient of determination (*R* ^2^) and variance in the YC. They were then evaluated on the ADOPIC baseline population to measure its impact on the separation between HC, MCI, and AD, assessed using Cohen *d*, and its correlation with MMSE. The separation between HC, MCI, and AD was also evaluated using the measures of longitudinal rate of change. Last, Spearman *ρ* was used to assess the correlation between the baseline CL and rate of change (CL/Yr).

For comparison, the same experiments were also conducted with each tracer quantified using its own tracer‐specific mask.

## 3 RESULTS

For each tracer, the number of matched HC/AD pairs were as follows: *N* = 147 for FBP, *N* = 22 for FBB, *N* = 10 for FLT, *N* = 42 for NAV, and *N* = 63 for PiB. Demographics for each of the groups are provided in Table 1. As per design, there was no difference in age or sex between the matched HC and AD participants within each tracer group. Additionally, there was no difference in age, sex, MMSE, or CDR between the HC participants selected for each tracer. There were, however, significant differences in CL between the HC participants across tracers, with CL in the FLT HC being higher than those in the HC from the other tracers. There was no difference in age, sex, MMSE, CDR, or CL between the AD participants selected for each tracer.

**TABLE 1:** Demographics of the ADOPIC participants selected to build the universal mask statistically. Bold font indicates statistical significance.

Tracer		PIB	FBP	FBB	FLT	NAV	*P*‐value
N pairs		63	147	22	10	42	
Sex (% female)	HC	0.44	0.5	0.23	0.4	0.48	0.21
	AD	0.44	0.5	0.23	0.4	0.48	0.21
	*P*‐value	1	1	1	1	1	
Age (years)	HC	75.4(7.9)	76.5(7.5)	75.4(5.8)	75.0(4.1)	72.9(8.5)	0.1
	AD	75.5(8.1)	76.5(8.0)	75.9(6.5)	75.1(4.3)	72.6(8.7)	0.11
	*P*‐value	0.939	0.945	0.8	0.987	0.909	
MMSE	HC	29.3(0.8)	29.3(0.8)	29.0(0.8)	28.9(0.9)	29.3(0.8)	0.28
	AD	22.1(1.4)	22.1(1.4)	22.2(1.6)	21.5(1.1)	22.1(1.4)	0.75
	*P*‐value	**<0.001**	**<0.001**	**<0.001**	**<0.001**	**<0.001**	
	Effect size	6.41	6.57	5.69	8.61	6.6	
CDR (% at 0/0.5/1/2)	HC	100/0/0/0	100/0/0/0	100/0/0/0	100/0/0/0	100/0/0/0	1
	AD	0/46/52/2	0/31/66/3	0/41/50/9	0/20/70/10	0/50/43/7	0.26
	*P*‐value	**<0.001**	**<0.001**	**<0.001**	**<0.001**	**<0.001**	
	Effect size	3.87	4.05	3.09	4.06	2.93	
Centiloid	HC	−1.6(5.6)	−3.6(10.6)	−3.1(11.0)	8.3(3.9)	−0.5(4.8)	**<0.001**
	AD	90.1(26.8)	87.5(29.3)	88.7(33.1)	102.1(34.3)	98.3(36.6)	0.22
	*P*‐value	**<0.001**	**<0.001**	**<0.001**	**<0.001**	**<0.001**	
	Effect size	4.84	4.17	3.98	4.5	3.92	

Abbreviations: AD, Alzheimer's disease; ADOPIC, Alzheimer's Dementia Onset and Progression in International Cohorts; CDR, Clinical Dementia Rating; FBB, ^18^F‐florbetaben; FBP, ^18^F‐florbetapir; FLT, ^18^F‐flutemetamol; HC, healthy control; MMSE, Mini‐Mental State Examination; NAV, ^18^F‐NAV4694; PIB, ^11^C Pittsburgh compound B.

**FIGURE 1:**
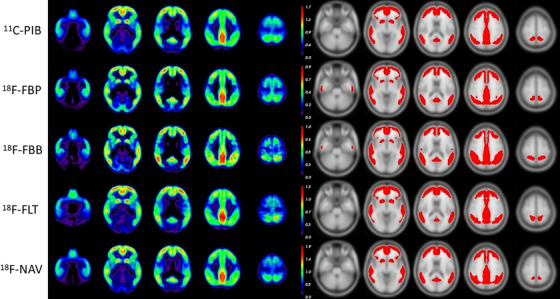
Difference images (AD‐HC) for all five tracers (with color‐scale maximum set at twice the optimal threshold value) and their corresponding masks. AD, Alzheimer's disease; ^11^C‐PiB, Pittsburgh compound B; FBB, ^18^F‐florbetaben; FBP, ^18^F‐florbetapir; FLT, ^18^F‐flutemetamol; HC, healthy control; NAV, ^18^F‐NAV4694

The difference image of each tracer and the corresponding mask that maximizes the overlap with the standard CL mask are presented in Figure 1. The corresponding thresholds and Dice scores are presented in Table 2. For each tracer, the colormap maximum was set at twice each tracer's respective threshold, giving them comparable dynamic range. Visually, there was a very high similarity between each tracer's difference image. The difference images for FBB and FLT were noisier compared to the other tracers, which was likely due to the lower number of scans included. The optimal threshold on the difference images was the highest for NAV, followed by PiB, FLT, FBB, and FBP. The highest overlap with the standard mask was obtained when using PiB, and the lowest with FLT and FBB. The pair‐wise masks comparisons presented in Table S1 in supporting information show that all masks have good overlap with each other (min Dice = 0.73).

**TABLE 2:** Optimal threshold (in SUVR) on each tracer's difference image and the corresponding dice score of the resulting tracer specific mask compared to the standard centiloid mask.

Tracer	Threshold	Dice
PIB	0.86	0.83
FBP	0.44	0.79
FBB	0.51	0.74
FLT	0.67	0.75
NAV	0.96	0.80

Abbreviations: FBB, ^18^F‐florbetaben; FBP, ^18^F‐florbetapir; FLT, ^18^F‐flutemetamol; NAV, ^18^F‐NAV4694; PIB, ^11^C Pittsburgh compound B.

**FIGURE 2:**
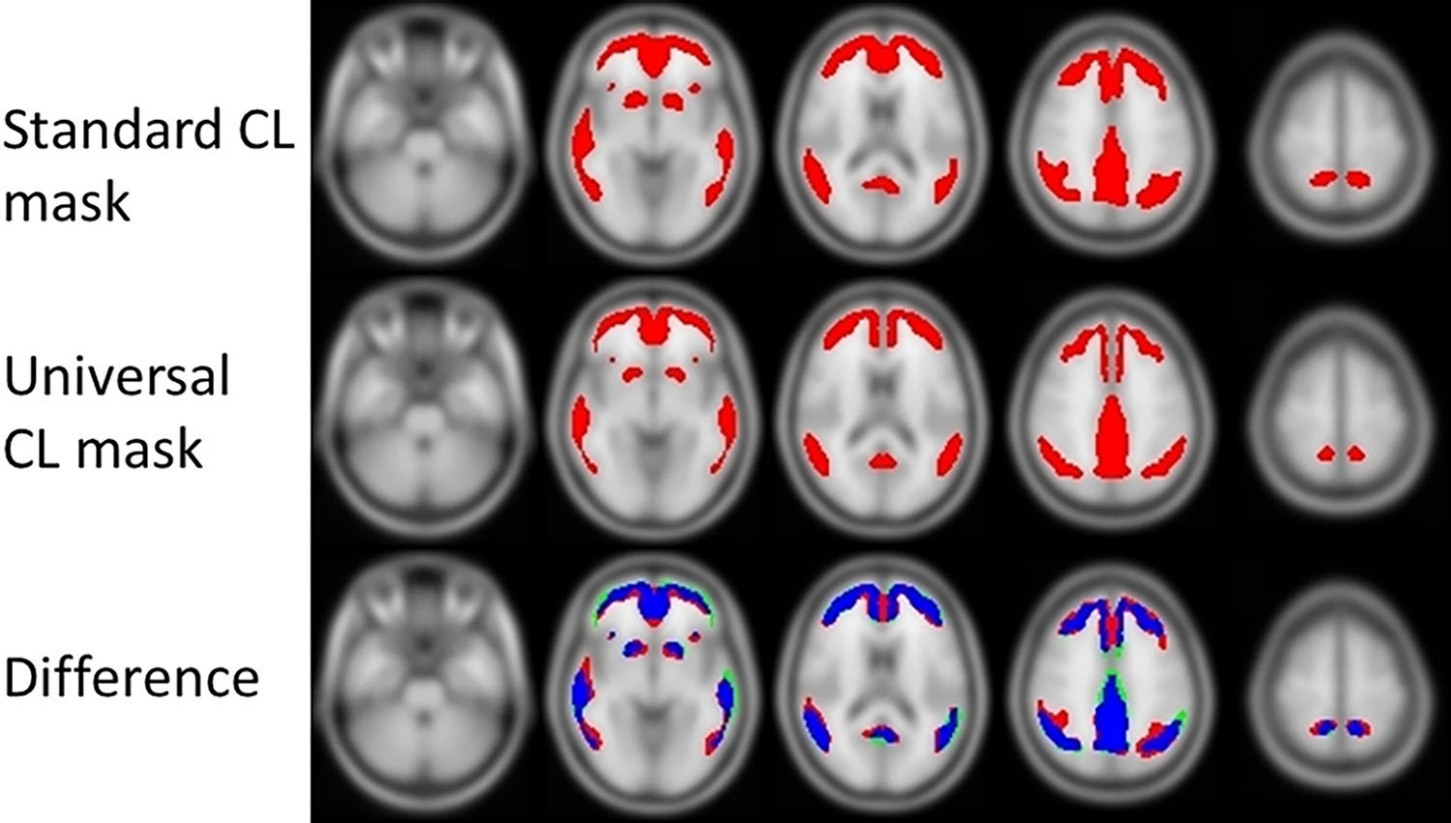
Standard and universal CL masks along with their difference (blue: common to both masks, red: only in standard mask, green: only in universal mask). CL, Centiloid;

The universal mask, defined as the intersection of all five masks is presented in Figure 2 along with the standard mask, and their overlaps and differences. There is a good overlap between the universal and standard mask (Dice = 0.74). The universal mask was, however, slightly narrower than the standard mask, especially in the frontal lobe, resulting in a 26% smaller volume.

The variance in the GAAIN YC and the correlation between the ^18^F‐Tracer/^11^C‐PiB pairs are presented in Table 3. The variance in the YC CLs was systematically lower using the universal mask compared to using the standard mask for all tracers (3.4% lower on average). The ^18^F‐Tracer/^11^C‐PiB correlations in the head‐to‐head subsets were also slightly higher when using the universal mask (0.24% higher on average).

**TABLE 3:** CL variance in the young controls and correlation (*R* ^2^) between each pair of 18F‐tracer and their corresponding ^11^C‐PIB using both the standard CL mask and the universal CL mask (lower variance and higher *R* ^2^ are marked in bold font).

Tracer	Variance YC (standard mask)	Variance YC (universal mask)	Correlation (*R* ^2^) with PIB (standard mask)	Correlation (*R* ^2^) with PIB (universal mask)
PIB	4.39	**4.19**	*NA*	*NA*
FBP	9.04	**8.67**	0.898	**0.902**
FBB	6.51	**6.16**	0.956	**0.959**
FLT	7.15	**7.01**	0.965	**0.967**
NAV	4.11	**4.06**	0.987	0.987

Abbreviations: CL, Centiloid; FBB, ^18^F‐florbetaben; FBP, ^18^F‐florbetapir; FLT, ^18^F‐flutemetamol; NAV, ^18^F‐NAV4694; PIB, ^11^C Pittsburgh compound B; YC, young control.

Using each tracer's specific mask did not reduce the variance in the YC compared to the universal mask (Table S2 in supporting information). While it improved the ^18^F‐Tracer/^11^C‐PiB correlations for FBP and NAV, it was decreased for FBB and FLT (Table S3 in supporting information).

The mean baseline CL and rate of changes are presented in Table 4, along with the group separation and correlation with MMSE in the ADOPIC dataset. The differences in CL at baseline between the standard and universal masks were < 1% for each tracer, and ≈1% for each clinical group. Using the universal mask on the ADOPIC dataset led to a slightly higher effect size at baseline between HC and MCI as well as HC and AD. The differences in effect size were, however, quite small (< 1%). The annualized rate of CL/Yr was slightly higher in the HC (+0.8%) and MCI (+2.2%) when using the universal mask, but lower in the AD (−5%). While the universal mask led to a higher effect size between HC and AD (+10%), it did not improve the separation between HC and MCI (−13%). Similarly, the correlation between CL and MMSE at baseline did not improve when using the universal mask, although the difference was < 0.5%.

**TABLE 4:** Mean CL at baseline and rate of CL change per year for each mask along with the corresponding group separation between the clinical groups, as well as correlation with MMSE for both the standard CL mask and the universal CL mask (higher effect size and higher *R* ^2^ are marked in bold font).

Measure	Target	Standard mask	Universal mask	Difference (%)
Mean (Std) baseline CL	HC	17.1 (31.6)	16.9 (32.1)	−1.07
	MCI	46.1 (48.8)	46.6 (49.5)	1.02
	AD	74.6 (47.7)	75.4 (47.9)	1.01
	PIB	25.6 (39.2)	25.7 (39.6)	0.21
	FBP	32.1 (44.5)	32.3 (45.3)	0.59
	FBB	28.4 (40.3)	28.2 (40.8)	−0.68
	FLT	36.4 (41.4)	36.2 (42.1)	−0.39
	NAV	54.5 (56.9)	54.7 (57.7)	0.45
Baseline CL effect size	HC vs. MCI	0.707	**0.712**	0.71
	HC vs. AD	1.422	**1.432**	0.70
Mean (Std) longitudinal CL/year	HC	1.59 (3.53)	1.6 (3.54)	0.81
	MCI	1.39 (3.94)	1.42 (3.92)	2.28
	AD	0.99 (5.11)	0.95 (4.95)	−4.98
Longitudinal CL/year effect size	HC vs. MCI	**0.052**	0.047	−9.62
	HC vs. AD	0.135	**0.152**	12.59
Correlation with baseline CL (*R* ^2^)	MMSE	**0.1484**	0.1479	−0.34

Abbreviations: AD, Alzheimer's disease; CL, Centiloid; FBB, ^18^F‐florbetaben; FBP, ^18^F‐florbetapir; FLT, ^18^F‐flutemetamol; HC, healthy control; MCI, mild cognitive impairment; MMSE, Mini‐Mental State Examination; NAV, ^18^F‐NAV4694.

It should also be noted that both sets of CL values were highly correlated, with a *R* ^2^ = 0.999 between the CLs obtained using the standard and universal masks (Figure S1a in supporting information). Similarly, the derived rates of change were highly correlated with *R* ^2^ = 0.996 (Figure S1b).

Last, when plotting the CL/Yr against baseline CL (Figure S2 in supporting information), the universal mask led to a slightly higher Spearman rank coefficient (*ρ* = 0.286) compared to using the standard mask (*ρ* = 0.271).

Using the tracer‐specific masks did not improve the effect size at baseline compared to using the universal mask (Table S4 in supporting information). While the separation between HC and AD using the annualized rate of CL/Yr increased, it got worse between HC and MCI. The correlation between CL and MMSE at baseline was slightly improved.

## 4 DISCUSSION

We have proposed a novel and tracer‐unbiased universal CL mask based on the five most commonly used Aβ tracers. This new mask is built as the intersection of the masks derived from the AD–HC difference images derived from each tracer, and therefore ensures that only regions where all five tracers measure Aβ are included. By defining the threshold based on the overlap with the standard mask, we also ensured that each mask has a similar extent to that of the standard mask.

Our matching procedure ensured that there were no differences in age or sex between the matched pairs of AD and HC. There were also no differences in age, sex, MMSE, or CDR between the HC (and AD) selected for each tracer, meaning that the HC (and AD) groups selected for each tracer were comparable based on these metrics.

Visually, all five different images showed very similar patterns of retention, indicating that all five tracers presented the same regional distribution. It should also be noted that while the FBB and FLT difference images tended to be noisier, this was primarily due to those two tracers having a much smaller number of pairs (10 for FLT and 22 for FBB) compared to the other tracers. The computed thresholds on each tracer's AD–HC difference image ranged from 0.44 to 0.96 SUVR, reflecting differences in the dynamic range for each tracer, with NAV having the highest threshold, and hence the highest dynamic range, followed by PiB, FLT, FBB, with FBP having the smallest one. It should, however, be noted that the HC selected for FLT had higher CL compared to the other tracers, which could potentially contribute to its lower dynamic range. The resulting masks also showed good concordance across tracers.

The universal mask was narrower than the standard mask, resulting in a sampling that avoids more cerebrospinal fluid (CSF) and white matter. Nevertheless, the standard and universal mask had a good overlap, with a Dice of 0.74.

Using the universal mask on the GAAIN calibration dataset led to a smaller variance in the YC and improved correlation between each head‐to‐head ^11^C‐PiB/^18^F‐Tracer datasets. As stated earlier, less sampling of white matter and CSF by the universal mask might explain the reduced variance in the YC; only sampling regions that are common to all five tracers also likely helped to improve the correlations in the paired dataset.

In the ADOPIC dataset, while using the universal mask increased the group separation between HC/MCI and HC/AD at baseline, those increases were very small (< 1%). The results were also mixed when using the longitudinal rate of change, increasing the HC/AD group separation, but decreasing the HC/MCI one.

As there is no ground truth for Aβ semi‐quantification, it can be difficult to assess the improvement given by using the universal mask, if any. While the results presented in this study indicate that a consensus universal mask can improve tracer agreement, it does not necessarily translate in improved quantification on a separate dataset, based on our chosen metrics. What those results, however, indicate is that using both the universal and standard masks led to very similar results, and the differences tended to be minimal. This supports that the current standard CL mask is suitable for the quantification of all Aβ tracers, and a universal neocortical mask is likely not needed. In other words, the benefits of the universal mask are too small and not sufficiently consistent to justify departing from the standard mask. Similarly, using tracer‐specific masks did not lead to large or systematic improvements over the standard mask.

One of the limitations of this study is that we used images smoothed to a uniform 8 mm point spread function (PSF), therefore losing some of the high resolution provided by more recent scanners. This was unfortunately unavoidable as different tracers were used on different scanners. Using the raw images instead of the smoothed images could have introduced differences in each tracer's mask that could reflect the scanner's sharpness, instead of the tracer's binding. Using images smoothed to a uniform PSF ensures that such bias is minimized. As newer scanners with higher resolution are increasingly being used in the clinic (e.g., Siemens Biograph Vision), a higher resolution mask might better exploit the higher contrast and sensitivity that those scanners provide, which could potentially lead to earlier detection of amyloid and more sensitive measures of temporal change. Last, this work did not explore different reference regions. Previous work using FBP has shown that the CL whole cerebellum might not be optimal for longitudinal^21^ or even cross‐sectional analysis.^7^ Future work looking at the optimal reference region for each tracer is therefore warranted.

## 5 CONCLUSIONS

The universal CL mask led to an increase in inter‐tracer agreement and group separation. Those increases were, however, relatively small indicating that a universal mask is not required, and that the existing standard CL mask is suitable for the quantification of all Aβ tracers.

## CONFLICT OF INTEREST STATEMENT

Chris Rowe has received research grants from Piramal Imaging, GE Healthcare, Cerveau, Astra Zeneca, and Biogen. Victor Villemagne is and has been a consultant or paid speaker at sponsored conference sessions for Eli Lilly, Piramal Imaging, Life Molecular Imaging, GE Healthcare, Abbvie, Lundbeck, Shanghai Green Valley Pharmaceutical Co Ltd, IXICO, and Hoffmann La Roche. The other authors have nothing to disclose.

## CONSENT STATEMENT

All AIBL participants gave written consent for publication of de‐identified data. All ADNI participants signed written informed consent for participation in the ADNI, as approved by the institutional revies board at each participating center. All OASIS‐3 participants consented to the use of their data by the scientific community and data sharing terms have been approved by the Washington University Human Research Protection Office.

## Supporting information

Supporting Information

## References

[1] Battle MR , Pillay LC , Lowe VJ , et al. Centiloid scaling for quantification of brain amyloid with [18F]flutemetamol using multiple processing methods. EJNMMI Res. 2018;8:107. doi:10.1186/s13550-018-0456-7 30519791 PMC6281542

[2] Klunk WE , Koeppe RA , Price JC , et al. The Centiloid Project: standardizing quantitative amyloid plaque estimation by PET. Alzheimer Dement. 2015;11:1‐15. doi:10.1016/j.jalz.2014.07.003 PMC4300247 25443857

[3] Navitsky M , Joshi AD , Devous MD , et al. Conversion of amyloid quantitation with florbetapir SUVR to the centiloid scale. Alzheimer Dement. 2016;12:P25‐P26. doi:10.1016/j.jalz.2016.06.032 30006100

[4] Rowe CC , Doré V , Jones G , et al. 18F‐Florbetaben PET beta‐amyloid binding expressed in Centiloids. Eur J Nucl Med Mol Imaging. 2017;44:2053‐2059. doi:10.1007/s00259-017-3749-6 28643043 PMC5656696

[5] Rowe CC , Jones G , Doré V , et al. Standardized expression of 18F‐NAV4694 and 11C‐PiB β‐Amyloid PET results with the centiloid scale. J Nucl Med. 2016;57:1233‐1237. doi:10.2967/jnumed.115.171595 26912446

[6] Bourgeat P , Dore V , Ames D , et al. Non‐negative matrix factorisation improves Centiloid robustness in longiyudinal studies. 2021;226. https://www.sciencedirect.com/science/article/pii/S1053811920310788 10.1016/j.neuroimage.2020.117593 PMC8049633 33248259

[7] Bourgeat P , Doré V , Burnham SC , et al. β‐amyloid PET harmonisation across longitudinal studies: application to AIBL, ADNI and OASIS3. Neuroimage. 2022;262:119527. doi:10.1016/j.neuroimage.2022.119527 35917917 PMC9550562

[8] Pegueroles J , Montal V , Bejanin A , et al. AMYQ: an index to standardize quantitative amyloid load across PET tracers. Alzheimer Dement. 2021. doi:10.1002/alz.12317. n/a. PMC8519100 33797846

[9] Leuzy A , Lilja J , Buckley CJ , et al. Derivation and utility of an Aβ‐PET pathology accumulation index to estimate Aβ load. Neurology. 2020;95:e2834‐e2844. doi:10.1212/WNL.0000000000011031 33077542 PMC7734735

[10] Whittington A , Gunn RN . Amyloid load: a more sensitive biomarker for amyloid imaging. J Nucl Med. 2019;60:536‐540. doi:10.2967/jnumed.118.210518 30190305 PMC6448463

[11] Villemagne VL , Doré V , Burnham SC , Masters CL , Rowe CC . Imaging tau and amyloid‐β proteinopathies in Alzheimer disease and other conditions. Nat Rev Neurol. 2018;14:225‐236. doi:10.1038/nrneurol.2018.9 29449700

[12] Juréus A , Swahn B‐M , Sandell J , et al. Characterization of AZD4694, a novel fluorinated Aβ plaque neuroimaging PET radioligand. J Neurochem. 2010;114:784‐794. doi:10.1111/j.1471-4159.2010.06812.x 20477945

[13] Ni R , Gillberg P‐G , Bergfors A , Marutle A , Nordberg A . Amyloid tracers detect multiple binding sites in Alzheimer's disease brain tissue. Brain. 2013;136:2217‐2227. doi:10.1093/brain/awt142 23757761

[14] Ellis KA , Bush AI , Darby D , et al. The Australian Imaging, Biomarkers and Lifestyle (AIBL) study of aging: methodology and baseline characteristics of 1112 individuals recruited for a longitudinal study of Alzheimer's disease. Int Psychogeriatr. 2009;21:672‐687. doi:10.1017/S1041610209009405 19470201

[15] LaMontagne PJ , Benzinger TL , Morris JC , et al. OASIS‐3: longitudinal neuroimaging, clinical, and cognitive dataset for normal aging and Alzheimer disease. MedRxiv 2019:2019.12.13.19014902. doi:10.1101/2019.12.13.19014902

[16] Petersen RC , Aisen PS , Beckett LA , et al. Alzheimer's Disease Neuroimaging Initiative (ADNI). Neurology. 2010;74:201‐209. doi:10.1212/WNL.0b013e3181cb3e25 20042704 PMC2809036

[17] McKhann G , Drachman D , Folstein M , Katzman R , Price D , Stadlan EM . Clinical diagnosis of Alzheimer's disease: report of the NINCDS‐ADRDA Work Group under the auspices of Department of health and human services task force on Alzheimer's Disease. Neurology. 1984;34:939‐944. doi:10.1212/wnl.34.7.939 6610841

[18] McKhann GM , Knopman DS , Chertkow H , et al. The diagnosis of dementia due to Alzheimer's disease: recommendations from the National Institute on Aging‐Alzheimer's association workgroups on diagnostic guidelines for Alzheimer's disease. Alzheimer Dement. 2011;7:263‐269. doi:10.1016/j.jalz.2011.03.005 PMC3312024 21514250

[19] Van der Kall LM , Truong T , Burnham SC , et al. Association of β‐amyloid level, clinical progression, and longitudinal cognitive change in normal older individuals. Neurology. 2021;96:e662‐e670. 33184233 10.1212/WNL.0000000000011222 PMC7884996

[20] Dice L . Measures of the amount of ecologic association between species. Ecology. 1945;26:297‐302.

[21] Landau SM , Fero A , Baker SL , et al. Measurement of longitudinal β‐amyloid change with 18F‐florbetapir PET and standardized uptake value ratios. J Nucl Med. 2015;56:567‐574. doi:10.2967/jnumed.114.148981 25745095 PMC5313473

