# Supplementary material for: A universal neocortical mask for Centiloid quantification
Source: Alzheimers Dement (Amst). 2023 Jul 21;15(3):e12457. doi: 10.1002/dad2.12457 (PMC10363815; doi:10.1002/dad2.12457)
Supplement: Supplementary file 1 — Supporting Information [file DAD2-15-e12457-s001.docx]

# ­­­Supplementary materials

Supplementary Table 1. Dice score between each pair of tracer specific masks, along with the Standard and Universal Centiloid mask.

|  | **PIB** | **FBP** | **FBB** | **FLT** | **NAV** | **Universal** | **Standard** |
| --- | --- | --- | --- | --- | --- | --- | --- |
| **PIB** | 1 | 0.87 | 0.80 | 0.83 | 0.91 | 0.80 | 0.83 |
| **FBP** | 0.87 | 1 | 0.82 | 0.77 | 0.85 | 0.79 | 0.79 |
| **FBB** | 0.80 | 0.82 | 1 | 0.73 | 0.81 | 0.77 | 0.74 |
| **FLT** | 0.83 | 0.77 | 0.73 | 1 | 0.82 | 0.79 | 0.75 |
| **NAV** | 0.91 | 0.85 | 0.81 | 0.82 | 1 | 0.80 | 0.80 |
| **Mean** | 0.85 | 0.83 | 0.79 | 0.79 | 0.85 | 0.79 | 0.78 |

Supplementary Table 2. CL variance in the young controls using the Standard CL mask, the Tracer-specific mask and the Universal CL mask (lower variance and higher R^2^ are marked in bold font)

| **Tracer** | **Variance YC (Std mask)** | **Variance YC (Tracer-specific mask)** | **Variance YC (Universal mask)** |
| --- | --- | --- | --- |
| **PIB** | 4.39 | 4.41 | **4.19** |
| **FBP** | 9.04 | 9.36 | **8.67** |
| **FBB** | 6.51 | 6.45 | **6.16** |
| **FLT** | 7.15 | 7.13 | **7.01** |
| **NAV** | 4.11 | 4.18 | **4.06** |

Supplementary Table 3. Correlation (R^2^) between each pair of ^18^F-tracer and their corresponding ^11^C-PIB using the Standard CL mask, the Tracer-specific mask and the Universal CL mask (lower variance and higher R^2^ are marked in bold font)

| **Tracer** | **Correlation (R^2^) with PIB (Std mask)** | **Correlation (R^2^) with PIB (Tracer-specific mask)** | **Correlation (R^2^) with PIB (Universal mask)** |
| --- | --- | --- | --- |
| **FBP** | 0.898 | **0.906** | 0.902 |
| **FBB** | 0.956 | 0.954 | **0.959** |
| **FLT** | 0.965 | 0.964 | **0.967** |
| **NAV** | 0.987 | **0.988** | 0.987 |

Supplementary Table 4. Group separation at baseline and using the rate of CL change per year between the clinical groups, as well as correlation with MMSE for both the Standard CL mask, the Tracer specific mask and the Universal CL mask (higher effect size and higher R^2^ are marked in bold font)

| **Measure** | **Target** | **Standard Mask** | **Tracer*-*specific Mask** | **Universal Mask** |
| --- | --- | --- | --- | --- |
| **Baseline CL Effect size** | **HC vs MCI** | 0.707 | 0.710 | **0.712** |
|  | **HC vs AD** | 1.422 | 1.421 | **1.432** |
| **Longitudinal CL/Year Effect size** | **HC vs MCI** | **0.052** | 0.039 | 0.047 |
|  | **HC vs AD** | 0.135 | **0.171** | 0.152 |
| **Correlation with Baseline CL (R^2^)** | **MMSE** | 0.1484 | **0.1492** | 0.1479 |


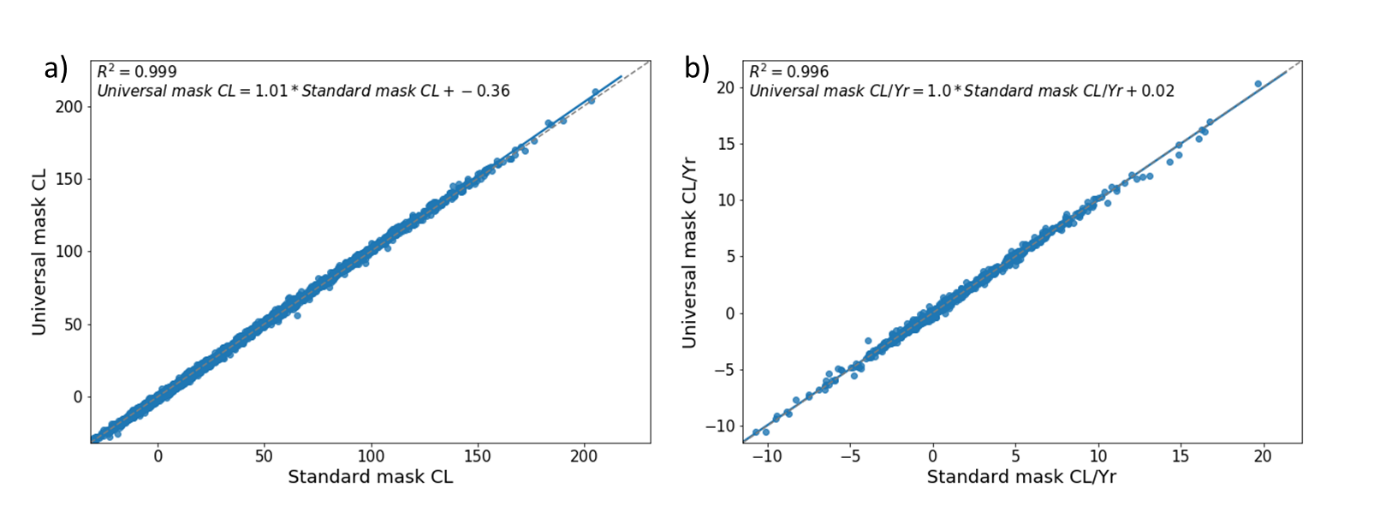


Supplementary Figure 1. Correlation between the Centiloids (a) and Centiloid/Yr (b) computed using the Universal mask, compared to the ones computed using the Standard mask in the ADOPIC study.


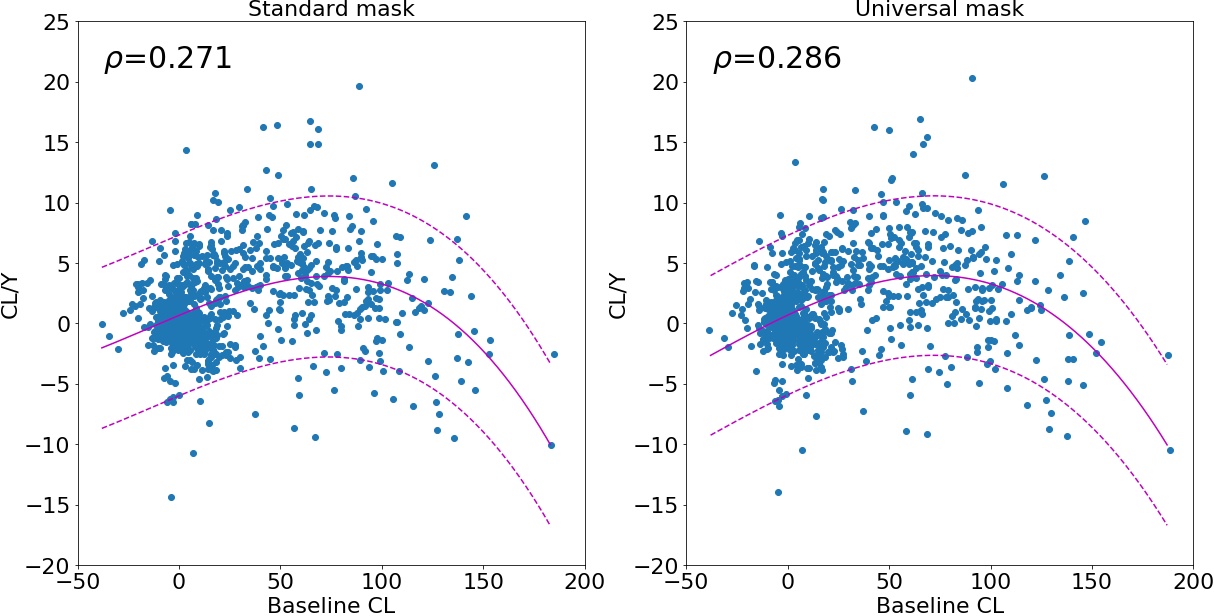


Supplementary Figure 2. Rate of CL change per year compared to baseline CL computed using the Standard mask (left) and Universal mask (right) in the ADOPIC study.
